# The Impact of Spermidine on C2C12 Myoblasts Proliferation, Redox Status and Polyamines Metabolism under H_2_O_2_ Exposure

**DOI:** 10.3390/ijms231910986

**Published:** 2022-09-20

**Authors:** Roberta Ceci, Guglielmo Duranti, Stefano Giuliani, Marianna Nicoletta Rossi, Ivan Dimauro, Stefania Sabatini, Paolo Mariottini, Manuela Cervelli

**Affiliations:** 1 Laboratory of Biochemistry and Molecular Biology, Department of Movement, Human and Health Sciences, Università degli Studi di Roma “Foro Italico”, 00135 Rome, Italy; 2 Department of Science, “Department of Excellence 2018–2022”, University of Rome “Roma Tre”, 00146 Rome, Italy; 3 Laboratory of Biology and Genetics, Department of Movement, Human and Health Sciences, Università degli Studi di Roma “Foro Italico”, 00135 Rome, Italy; 4 Neurodevelopment, Neurogenetics and Molecular Neurobiology Unit, IRCCS Fondazione Santa Lucia, Via del Fosso di Fiorano 64, 00143 Rome, Italy

**Keywords:** spermidine, polyamines homeostasis, glutathione, redox homeostasis, myoblasts proliferation

## Abstract

A central feature of the skeletal muscle is its ability to regenerate through the activation, by environmental signals, of satellite cells. Once activated, these cells proliferate as myoblasts, and defects in this process profoundly affect the subsequent process of regeneration. High levels of reactive oxygen species such as hydrogen peroxide (H_2_O_2_) with the consequent formation of oxidized macromolecules increase myoblasts’ cell death and strongly contribute to the loss of myoblast function. Recently, particular interest has turned towards the beneficial effects on muscle of the naturally occurring polyamine spermidine (Spd). In this work, we tested the hypothesis that Spd, upon oxidative challenge, would restore the compromised myoblasts’ viability and redox status. The effects of Spd in combination with aminoguanidine (Spd-AG), an inhibitor of bovine serum amine oxidase, on murine C2C12 myoblasts treated with a mild dose of H_2_O_2_ were evaluated by analyzing: (i) myoblast viability and recovery from wound scratch; (ii) redox status and (iii) polyamine (PAs) metabolism. The treatment of C2C12 myoblasts with Spd-AG increased cell number and accelerated scratch wound closure, while H_2_O_2_ exposure caused redox status imbalance and cell death. The combined treatment with Spd-AG showed an antioxidant effect on C2C12 myoblasts, partially restoring cellular total antioxidant capacity, reducing the oxidized glutathione (GSH/GSSG) ratio and increasing cell viability through a reduction in cell death. Moreover, Spd-AG administration counteracted the induction of polyamine catabolic genes and PA content decreased due to H_2_O_2_ challenges. In conclusion, our data suggest that Spd treatment has a protective role in skeletal muscle cells by restoring redox balance and promoting recovery from wound scratches, thus making myoblasts able to better cope with an oxidative insult.

## 1. Introduction

Skeletal muscle is a key tissue for maintaining the optimal energy expenditure of the whole organism, and therefore, the goal of preserving a healthy skeletal muscle mass is an important task to pursue throughout the entire life span to prevent/mitigate possible metabolic diseases. A central feature of the skeletal muscle is the ability to regenerate through the activation, by environmental signals, of stem cells (satellite cells) [1]. Once activated, satellite cells proliferate as myoblasts and then, after aligning and fusing, they form multinucleated cells that repair the damaged sections of the myofibers and maintain muscle mass [2,3]. Defects in the activation/proliferation of myoblasts profoundly affect the subsequent process of regeneration [4]. It is noteworthy to mention that a reduction in the regenerative potential of the muscle characterizes several physiopathological conditions, such as atrophy (muscle mass loss) and amyotrophic lateral sclerosis, that severely affect human health [5,6,7,8]. Reactive oxygen species (ROS), such as hydrogen peroxide (H_2_O_2_), exert a critical regulatory role on skeletal muscle function [9,10,11]. ROS levels from low to moderate are critical for cell signaling and regulation of gene expression; they act as signals for cell adaptation and are necessary for muscle growth [10,12]. On the contrary, high levels of ROS with the consequent formation of oxidized macromolecules may contribute to the loss of myoblast function, increase myoblast cell death and worsen muscle repair in aging, events that are associated with metabolic diseases like diabetes or muscle mass loss such as atrophy and sarcopenia [13]. It is also worth mentioning that many types of physical exercise, especially intense or unaccustomed, result in oxidative stress, as evidenced by increased biomarkers of oxidation in both skeletal muscle and blood [12,14,15]. Moreover, long periods of muscle disuse also promote ROS production in skeletal muscle fibers [16]. Nutritional strategies to mitigate the damage induced by high ROS levels to macromolecules are the subject of much ongoing research [17,18]. Recently, a particular interest has turned towards the beneficial effects of the naturally occurring polyamine spermidine (Spd) at the muscular level [19,20,21]. Spermidine, together with spermine (Spm) and their precursor putrescine (Put), is the organic polycation present in small quantities in every human, animal and plant cell. They are necessary for cell growth, renewal and metabolism in all eukaryotes [22]. 

**Figure 1:**
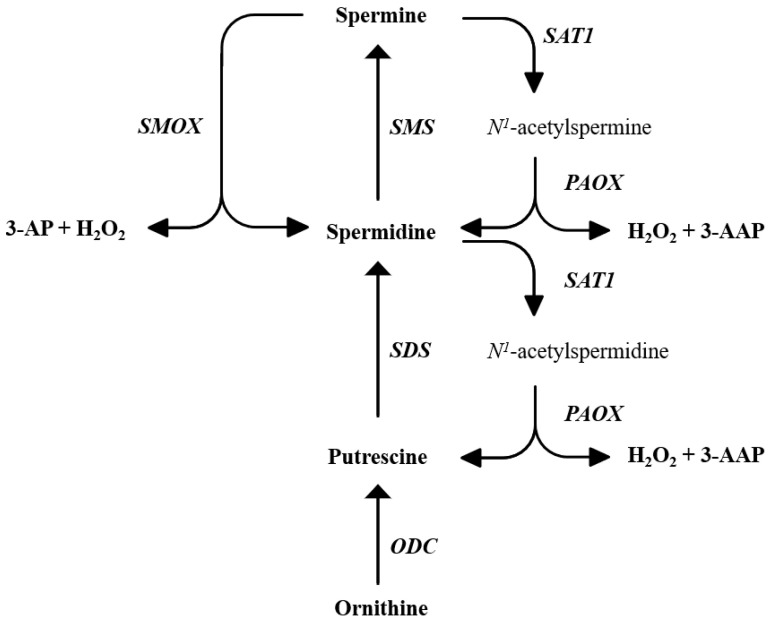
Schematic representation of polyamine metabolism showing enzyme networks and substrate interconversion pathways. PAOX: acetylpolyamine oxidase, ODC: ornithine decarboxylase, SMOX: spermine oxidase, SDS: spermidine synthase, SMS: spermine synthase, SAT1: spermine/spermidine N1-acetyl-transferase, 3-AP: 3-aminopropanal, 3-AAP: 3-acetoamidopropanal.

The biosynthetic and catabolic pathways of polyamines (PAs) in mammals are well described in many reviews [23,24,25,26]. Briefly, PA biosynthesis begins from the precursor ornithine, forming Put, and from here, Spd and Spm are formed by tightly regulated reactions. On the other hand, their catabolism involves the enzymes spermine oxidase (SMOX), spermine-/spermidine-N1-acetyltransferase 1 (SAT1) and polyamine oxidase (PAOX), which, as a whole, regulate PAs homeostasis [27] (Figure 1). Polyamines, among the other biological functions, act as antioxidants and free radical scavengers, therefore contributing to the oxidative balance of the cell [25,28,29]. 

As reported in microbiological studies, in Escherichia coli cells, PAs are the main scavengers of free radicals, and they also act as positive modulators of antioxidant genes when subjected to challenging conditions [30]. 

Moreover, PAs show antioxidant properties against hydrogen peroxide in red blood cells, decreasing hemolysis probably through the stabilization of lipids in the cytoplasmic membranes. Negatively charged phospholipids can react with positively charged amino groups of Pas, which can also protect DNA and RNA from oxidation damage induced by ROS [31]. 

However, on the one hand, they act as protectors against oxidative damage, but on the other, they serve as substrates for oxidation reactions that produce H_2_O_2_ intra- and extracellularly [32,33,34,35,36,37]. Thus, maintaining intracellular polyamine homeostasis is important to maintain an optimal cellular oxidative balance. 

Spermidine is turning out to be a molecule of great interest for the prevention or treatment of muscle diseases; in fact, a decrease in its cellular concentration has been related to skeletal muscle atrophy. In recent years, many studies have addressed the effects of Spd supplementation on muscle mass [2,38,39]. Evidence demonstrates that skeletal muscle atrophy in D-galactose-treated rats was reverted by Spd administration, particularly in combination with exercise. This effect involved the induction of autophagy and mitochondrial improvements [38]. The Spd-mediated ultrastructural and functional improvement of mitochondria from aged cardiac muscles and from skeletal muscle (stem) cells further supports the potential utility of Spd in the treatment of muscle-related disorders [39,40]. Although the strong association between Spd levels and muscle mass is becoming evident, the effects of Spd on counteracting oxidative stress, restoring redox balance as well as its effect on proliferation in skeletal muscle cells are a field quite unexplored so far. In this paper, we tested the hypothesis that Spd, upon oxidative challenge, would restore the compromised myoblast viability and redox status. Therefore, the effect of Spd in combination with aminoguanidine (Spd-AG), an inhibitor of bovine serum amine oxidase (BSAO), on murine C2C12 myoblasts treated with a mild dose of H_2_O_2_ was evaluated by analyzing: (i) myoblast viability and recovery from wound scratch; (ii) redox status and (iii) polyamine metabolism.

## 2. Results

### 2.1. Hydrogen Peroxide Treatment Reduces C2C12 Myoblast Viability and Total Antioxidant Capacity

Cell viability was assessed by a direct cell count through the trypan blue assay. This test allows one to distinguish dead cells from living cells. Cells were treated with a range of H_2_O_2_ (0.1–1 mM) for 24 h. 

**Figure 2:**
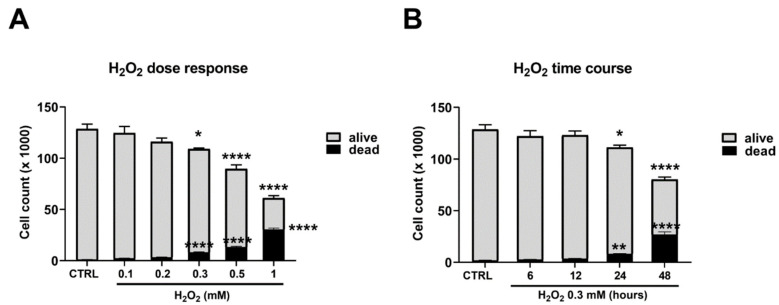
**H_2_O_2_ treatment and C2C12 myoblast viability.** (**A**) C2C12 myoblasts were treated with different H_2_O_2_ concentrations (0.1–1 mM) for 24 h; (**B**) C2C12 myoblasts were treated with H_2_O_2_ 0.3 mM for different times (6–48 h). Cell viability was assessed by the trypan blue exclusion assay. Results are expressed as the number of cells (×1000). Data presented are the mean ± SEM of three experiments, each performed in triplicate. One-way ANOVA was performed followed by Bonferroni’s multiple comparisons test * *p* < 0.05; ** *p* < 0.01; **** *p* < 0.0001 vs. CTRL.

We found that as the concentration of H_2_O_2_ increased, the number of viable cells decreased, with a concomitant rise in dead cells in a dose-dependent way (Figure 2, panel A). A concentration of 0.3 mM of H_2_O_2_ was then selected for all subsequent experiments.

The time course (6–48 h) showed a statistically significant decrease in the number of cells accompanied by an increase in dead cells after 24 h of treatment with a maximum at 48 h (Figure 2B). 

**Figure 3:**
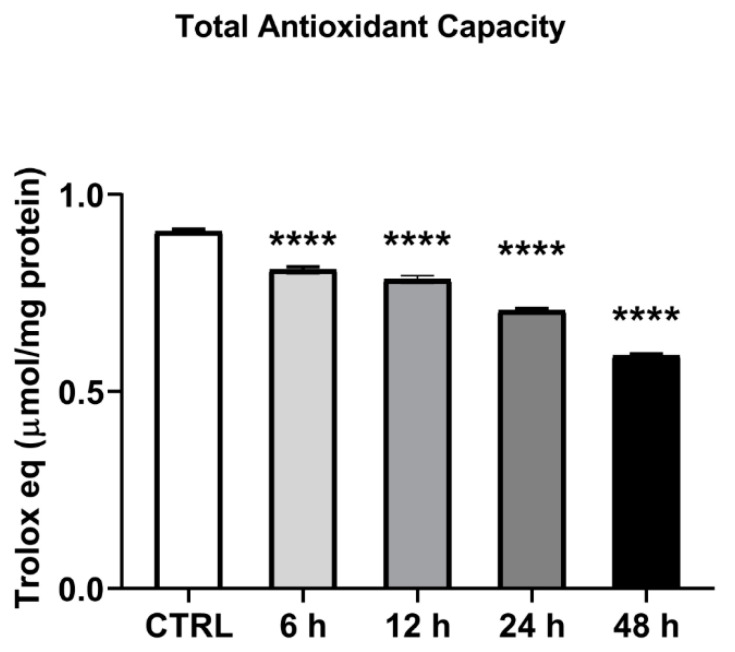
**H_2_O_2_ treatment and total antioxidant capacity.** C2C12 myoblasts were treated with H_2_O_2_ 0.3 mM for different times (6–48 h) and cell lysates were tested for TAC analysis. TAC levels were expressed as micromoles of Trolox equivalents/mg of protein tested. Data presented are the mean ± SEM of three experiments, each performed in triplicate. One-way ANOVA was performed followed by Bonferroni’s multiple comparisons test **** *p* < 0.0001 vs. CTRL.

The time-dependent (6–48 h) effect of H_2_O_2_ 0.3 mM on cellular total antioxidant capacity (TAC) was tested. A statistically significant decrease in TAC value was found up to a 35% reduction after 48 h of treatment (*p* < 0.01, Figure 3).

### 2.2. Hydrogen Peroxide Treatment Reduces Intracellular Polyamines Content

**Figure 4:**
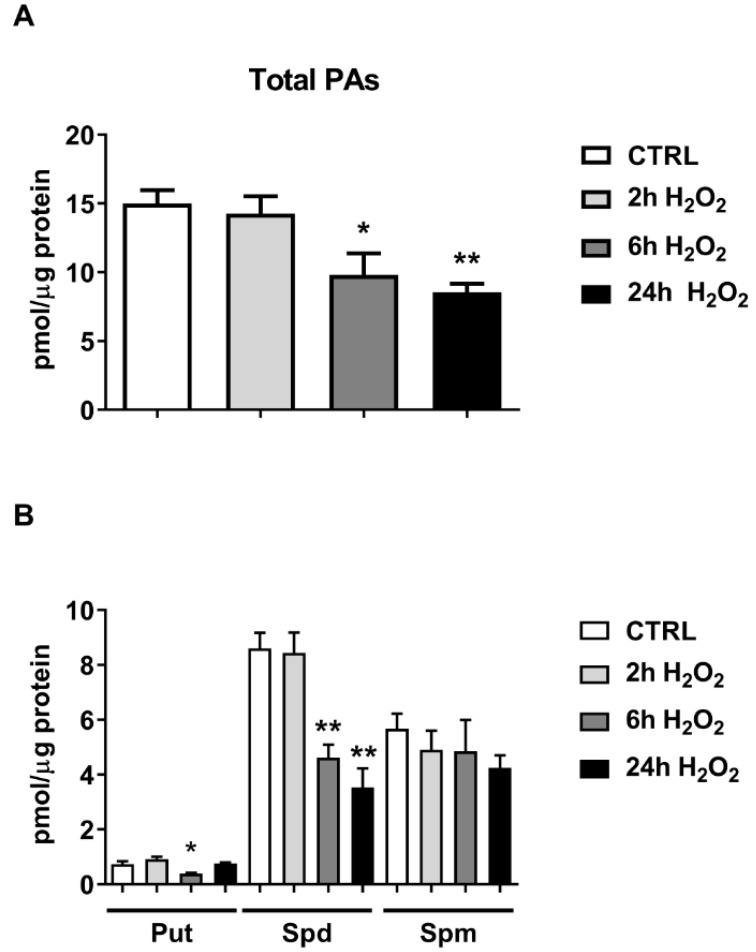
**Effect of H_2_O_2_ on C2C12 myoblast polyamine content.** C2C12 myoblasts were treated with H_2_O_2_ 0.3 mM for different times (2–24 h). (**A**) Evaluation of total polyamines content; (**B**) Evaluation of Put, Spd and Spm levels. Data presented are the mean ± SEM of three experiments, each performed in triplicate. One-way ANOVA was performed followed by Bonferroni’s multiple comparisons test * *p* < 0.05; ** *p* < 0.01 vs. CTRL.

The time-dependent (2–24 h) effect of H_2_O_2_ 0.3 mM on C2C12 PAs content was tested. Total PA content reveals a significant decrease after 6 and 24 h of H_2_O_2_ treatment (65% and 57% of CTRL, *p* < 0.05), as shown in Figure 4A.

The evaluation of Put, Spd and Spm levels showed a statistically significant decrease of 47% in Put content at 6 h (*p* < 0.05) and in Spd content at 6 h (53% of CTRL, *p* < 0.01) and 24 h (40% of CTRL, *p* < 0.01), Figure 4A. No statistically significant effect was found in Spm levels (Figure 4B). 

### 2.3. Effect of Spermidine and Aminoguanidine Treatment on C2C12 Myoblast Viability

**Figure 5:**
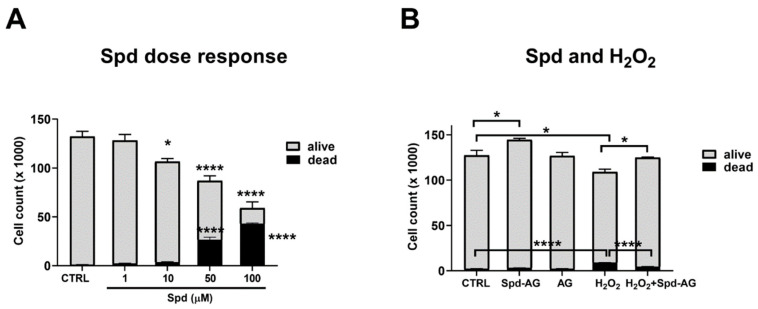
**Effect of Spd, AG, Spd-AG and H_2_O_2_ in single and combined treatments on C2C12 myoblast viability.** (**A**) C2C12 myoblasts were treated with different Spd concentrations (1–100 μM) for 24 h; (**B**) C2C12 myoblasts were treated with H_2_O_2_ 0.3 mM, Spd 10 μM and AG 1mM in various combinations for 24 h. Cell viability was assessed by the trypan blue exclusion assay. Results are expressed as the number of cells (×1000). Data presented are the mean ± SEM of three experiments, each performed in triplicate. One-way ANOVA was performed, followed by Bonferroni’s multiple comparison tests for panels A and B, respectively. * *p* < 0.05; **** *p* < 0.0001.

Previous studies reported that in vitro treatment with Spd caused a dose-dependent toxicity in different cell lines [41,42,43,44]. To evaluate this possible cytotoxic effect on myoblasts, cells were treated with a range of Spd (1–100 μM) for 24 h. We also found a dose-dependent toxic effect (*p* < 0.05) in our experimental model starting from the dose of 10 μM (*p* = 0.04, Figure 5A). Hence, to avoid this harmful effect, for the subsequent experiment, aminoguanidine (AG), an inhibitor of the diamino-oxidases normally present in fetal bovine serum, was used in combination with Spd to prevent its toxicity, as previously reported [45]. AG prevents the formation of toxic compounds derived from the oxidation of Spd by bovine serum oxidase.

For the subsequent experiments, H_2_O_2_ 0.3 mM, Spd 10 μM and AG 1 mM concentrations were chosen [45]. We found that AG administration for 24 h did not cause any statistically significant difference compared to the control. On the other hand, a combined treatment of Spd plus AG (Spd-AG) induced a statistically significant increase in cell count compared to the control (15%, *p* < 0.05, Figure 5B). Hydrogen peroxide treatment induced a decrease in the number of cells (*p* < 0.05) and an increase in cell death (*p* < 0.005) compared to the control (Figure 5B). Interestingly, Spd-AG recovered, in a statistically significant manner, the harmful effect that hydrogen peroxide induced (*p* < 0.05, Figure 5B).

### 2.4. Spermidine-AG Accelerates Scratch Wound Closure

To investigate the role played by our treatments on the migrative ability of C2C12 cells, confluent cells grown under proliferative conditions were scratched and wound closure was measured [46]. In detail, before each treatment, a wound of 1 mm was created in the culture plate, and after 24 h, the sulcus width was measured in control and treated cells.

**Figure 6:**
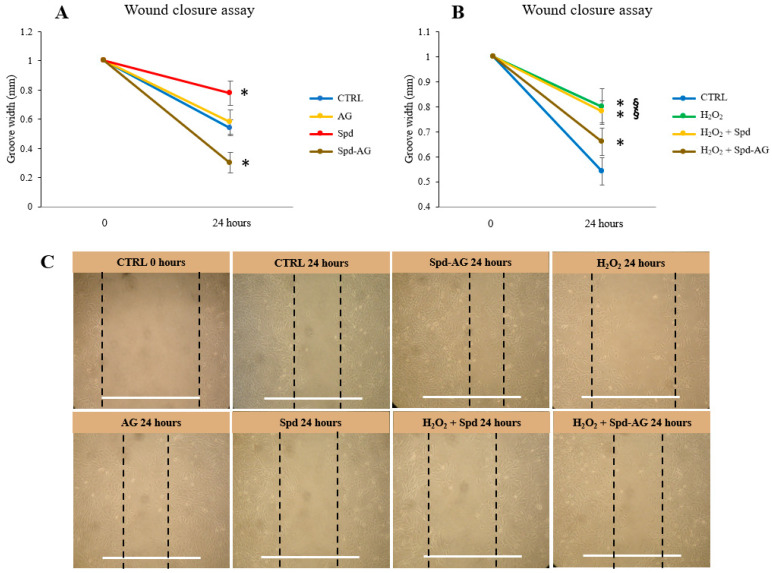
**Wound closure assay.** Before each treatment, a wound of 1 mm was created in the culture plate. Cells were treated with Spd 10 μM, AG 1 mM, H_2_O_2_ 0.3 mM and combined treatments or a vehicle for 24 h. The sulcus width was then measured in control and treated cells. (**A**) Effect of Spd, AG and Spd-AG. (**B**) Effect of H_2_O_2_ and combined treatment with Spd and Spd-AG. (**C**) Representative images of all treatments. White bars correspond to 1 mm. Data and representative images presented are the mean ± SD of three experiments, each performed in triplicate. One-way ANOVA was performed, followed by Bonferroni’s multiple comparison tests. * *p* < 0.05 vs. CTRL; § *p* < 0.05 vs. H_2_O_2_.

After 24 h, a reduction of 46% and 42% of wound width were observed for CTRL and AG samples, respectively. After Spd and H_2_O_2_ single treatment, a minor reduction was observed compared to CTRL 24 h; a 22% reduction (*p* < 0.01) was found in cells treated with Spd and a 20% wound reduction after H_2_O_2_ treatment (*p* < 0.01). Interestingly, a greater closure was observed after Spd-AG, in fact, a 70% reduction in wound width was found after treatment (*p* < 0.01) (Figure 6A,B).

The combined treatment of Spd-AG + H_2_O_2_ showed that the presence of Spd-AG allowed cells to increase the ability to close the wound (34% *p* < 0.01) compared to H_2_O_2_ alone (*p* < 0.01) or compared to Spd + H_2_O_2_ (*p* < 0.01) (Figure 6B,C).

### 2.5. Spermidine-AG Treatment Restores Redox Status and Total Antioxidant Capacity in C2C12 Myoblasts

The evaluation of glutathione homeostasis revealed significant differences in the GSH/GSSG ratio, a well-known marker of redox status, between the different treatments.

**Figure 7:**
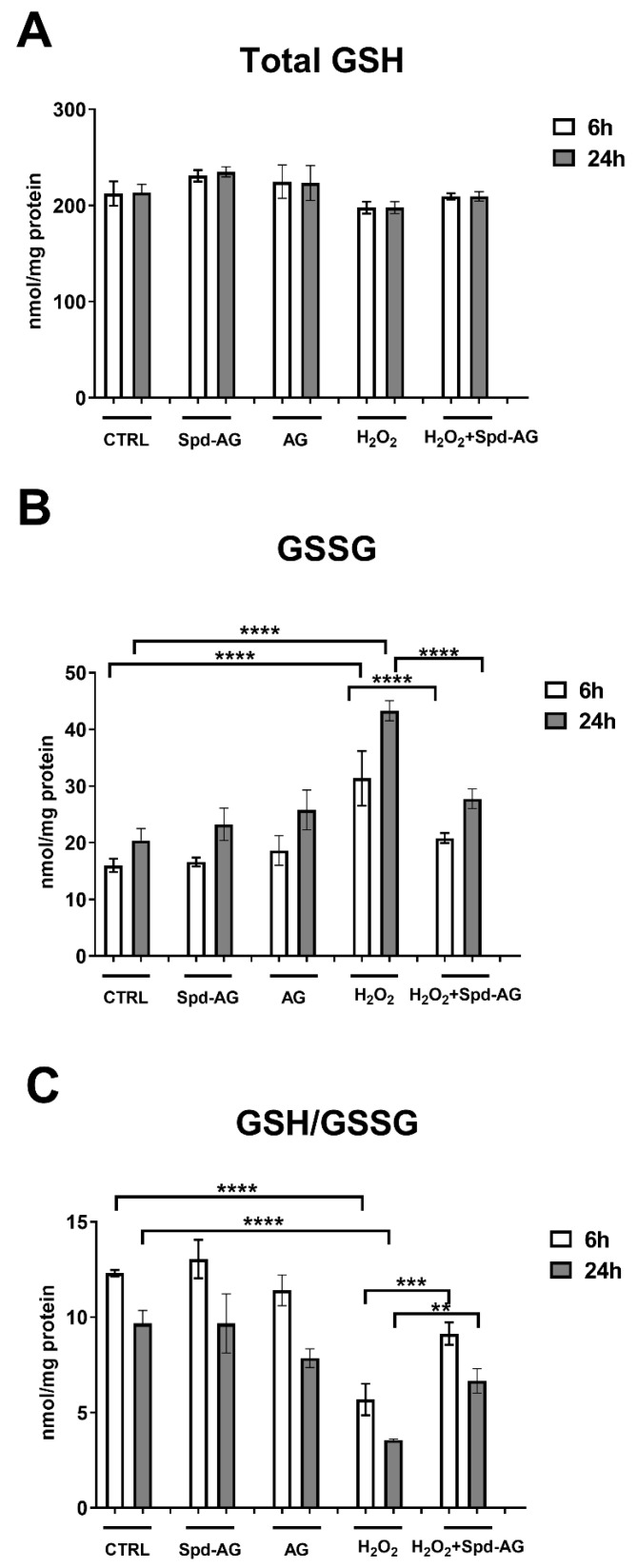
**Glutathione homeostasis evaluation.** (**A**) Total glutathione (tGSH); (**B**) oxidized glutathione (GSSG) and (**C**) reduced to oxidized glutathione ratio (GSH/GSSG) were evaluated in C2C12 myoblasts after 6 and 24 h in the presence of aminoguanidine (AG, 1 mM), spermidine (Spd, 10 μM), hydrogen peroxide (H_2_O_2_, 0.3 mM) and combined treatments. Data presented are the mean ± SEM of three experiments, each performed in triplicate. A two-way ANOVA test followed by Bonferroni’s multiple comparison test was performed. ** *p* < 0.01; *** *p* < 0.001; **** *p* < 0.0001.

The analysis of total glutathione levels (tGSH) revealed that there were essentially no statistically significant changes across all treatments (Figure 7A).

H_2_O_2_ treatment induced an increase in GSSG levels (193% *p* < 0.005) compared to control (Figure 7B). No statistically significant differences were found after AG or Spd-AG treatments. Interestingly, Spd-AG in combination with H_2_O_2_ prevents, in a statistically significant manner, the H_2_O_2_-induced increase in oxidized glutathione (*p* < 0.05, Figure 7). The evaluation of the ratio between reduced and oxidized glutathione (GSH/GSSG) showed a statistically significant reduction after H_2_O_2_ treatment (59%, *p* < 0.01) and the combined treatment with Spd-AG limitedly reversed (32%, *p* < 0.01), in a statistically significant manner, this decrease (*p* < 0.01, Figure 7C).

**Figure 8:**
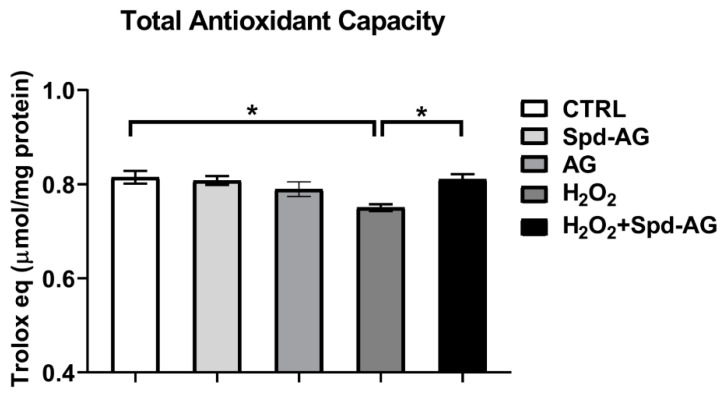
**Effect of Spd-AG and H_2_O_2_ on C2C12 myoblasts on total antioxidant capacity.** C2C12 myoblasts were treated with H_2_O_2_ 0.3 mM, AG, 1 mM, Spd, 10 μM and combined treatments for 24 h. Cell lysates. TAC was expressed as micromole Trolox equivalents/mg of protein tested. Data presented are the mean ± SEM of three experiments, each performed in triplicate. A one-way ANOVA followed by Bonferroni’s multiple comparisons test was performed. * *p* < 0.05.

The analysis of total antioxidant capacity showed a statistically significant reduction after 24 h H_2_O_2_ treatment (*p* < 0.01) and the combined treatment with Spd-AG was able to revert in a statistically significant manner this decrease (*p* < 0.01, Figure 8).

### 2.6. Spd-AG Treatment Counteracts H_2_O_2_-Induced Polyamines Imbalance

**Figure 9:**
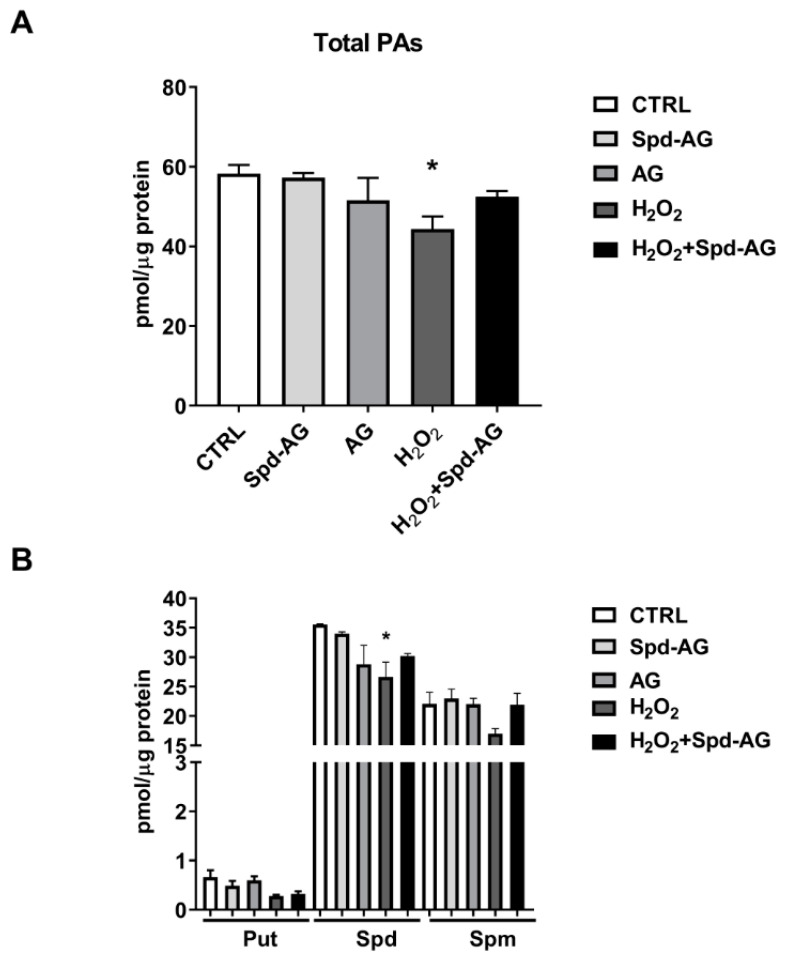
**Effect of Spd-AG and H_2_O_2_ on C2C12 myoblast polyamine content.** Cells were treated with Spd 10 μM, AG 1 mM, H_2_O_2_ 0.3 mM and combined treatments for 6 h. (**A**) Total polyamine content; (**B**) Put, Spd and Spm levels. Data presented are the mean ± SEM of three experiments, each performed in triplicate. One-way ANOVA was performed with Bonferroni’s multiple comparison test * *p* < 0.05.

A statistically significant decrease of 24% of the total PA content was found after H_2_O_2_ treatment (*p* < 0.05, Figure 9A), mirrored by a statistically significant decrease in Spd levels (75% of CTRL content, *p* < 0.05, Figure 9B). Treatment with AG, Spd-AG or the triple combination of Spd-AG-H_2_O_2_ did not exert any effect on the levels of Put, Spd and Spm (Figure 9B). 

### 2.7. Effect of Spd-AG and Hydrogen Peroxide Treatment on Polyamines Enzymatic Pathway

**Figure 10:**
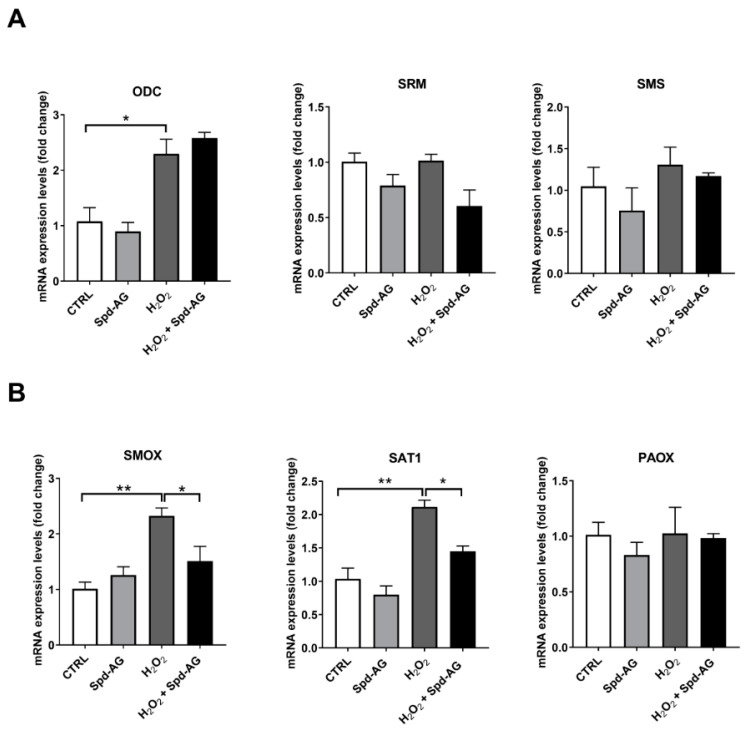
**Effect of Spd-AG and H_2_O_2_ on the expression level of polyamines enzymatic pathway.** C2C12 were treated with Spd 10μM in combination with AG 1 mM and/or H_2_O_2_ 0.3 mM for 6 h and RNA transcript expression of the polyamine enzymatic pathway was evaluated. qRT-PCR of biosynthetic enzymes (**A**) ornithine decarboxylase (ODC), spermidine synthase (SDS), spermine synthase (SMS), or catabolic enzymes (**B**) spermine oxidase (SMOX), spermine-/spermidine-N1-acetyltransferase 1 (SAT1) and polyamine oxidase (PAOX) amplified from C2C12 myoblasts were assayed. Data are calculated relative to the internal housekeeping gene (GAPDH) and are expressed as the mean fold change compared with control. Each value represents the mean ± SEM of three independent experiments, each performed in triplicate. One-way ANOVA followed by Bonferroni’s multiple comparisons test was performed * *p* < 0.05; ** *p* < 0.01.

The evaluation of the PA homeostasis enzymatic pathway was performed by qPCR analysis. In Figure 10, the expression levels of biosynthetic (Figure 10A) and catabolic enzymes (Figure 10B) have been analyzed.

Hydrogen peroxide treatment induced a statistically significant increase in ODC expression level (278% vs. CTRL *p* < 0.001), the addition of Spd-AG did not revert the increase. Spermidine synthase (SDS) and spermine synthase (SMS) were found unaffected after treatment (Figure 10A). 

A significant up regulation of spermine oxidase (SMOX) (210% vs. CTRL, *p* < 0.0001) and spermine-/spermidine-N1-acetyltransferase 1 (SAT1) (283% vs. CTRL, *p* < 0.001) was observed after 6 h H_2_O_2_ treatment. Interestingly, these increases were reverted by the combined treatment with Spd-AG.

## 3. Discussion

The polyamines Put, Spd and Spm are small, endogenously-produced polycations that play an important role in cell growth and differentiation [47,48]. Studies with inhibitors of polyamine synthesis and polyamine deficient mutants have demonstrated that these molecules are essential for cell growth [20,49,50,51]. Moreover, polyamines seem to play a role in protecting cells from ROS in different cell systems [52], and it has also been shown that depletion of endogenous cellular polyamines results in increased sensitivity to ROS produced by radiation exposure [53,54]. 

A goal of the study was focused on myoblast proliferation because its efficiency represents a key factor for optimal muscle regeneration. It must be recalled that muscle fibers are formed from the fusion of myoblasts that become terminally differentiated and exit the cell cycle. In adult life, the only mechanism of postnatal muscle growth is possible through the hypertrophy of existing fibers following the activation of the normally quiescent satellite cells, in a mechanism also necessary for the repair of damaged muscle fibers [1].

In this context, we used C2C12 myoblasts, derived from satellite cells, that are considered a good model to study in vitro muscle proliferation and differentiation [55,56,57]. 

They are commonly used to study muscle regeneration due to their ability to transition, upon adequate stimulus, from a proliferative phase into differentiated myofibers, similar to satellite cell behavior.

The present study showed that, in C2C12 myoblasts, a mild non-cytotoxic H_2_O_2_ treatment caused growth inhibition, perturbation in redox status and total antioxidant capacity, concomitantly with a decrease in intracellular total polyamines. Particularly, a statistically significant reduction of Spd was observed. Considering the importance of polyamines in cell survival, we treated myoblasts with different doses of spermidine in an attempt to reestablish their physiological intracellular content and, in such a way, possibly restore the redox status and proliferative efficiency altered by H_2_O_2_ challenge. We have found that in our model of muscle cells, Spd has a cytotoxic effect along with an inhibitory effect on cell growth.

Many studies have reported that treatment with Spd in vitro can cause dose-dependent toxicity in different cell lines. The explanation for this observation is that it can be oxidized by several intra- and extracellular oxidases. It is particularly relevant that bovine serum amine oxidases are found in bovine serum, which is normally used in culture cells for its many components necessary for cell growth [41,42,43,44]. 

The BSAO can oxidize Spd and Spm to aminoaldehydes and H_2_O_2_, which can both cause cytotoxicity [25], as well as the activation of cellular stress responses [35], and can overall obscure the biologically relevant effects induced by the intact non-oxidized molecule. 

Since C2C12 myoblasts are normally supplemented with bovine calf serum that is essential for optimal growth, to eliminate the confounding effect deriving from Spd oxidation, we performed the experiments using AG, a well-tolerated inhibitor of BSAO. Supplementation of AG is nontoxic to cells, and it can be used safely in millimolar concentrations [41,42,43,44,45]. AG treatment did not show any effect on the parameters analyzed in our experimental model. Studies from literature performed in vivo and on other cellular models showed that spermidine effectively plays a role in promoting proliferation [58,59,60] and as an antioxidant molecule [61,62,63], but our data showed that these effects are only detectable when Spd is in combination with AG. 

Interestingly, Spd-AG treatment increased proliferation as shown by the accelerated recovery from wound scratches when compared with control cultures. The wound scratch assays simulate a damaging situation in which myoblasts present at the injury site (i.e., mechanically induced) are evaluated for their migration/proliferation capacity when stimulated. The assay is meant to mimic what happens during the degeneration/inflammation phase that precedes muscle regeneration, a phenomenon that requires that satellite cells/myoblasts migrate to the site of injury where they proliferate before the differentiation process.

The presence of Spd-AG in the combined treatment with H_2_O_2_ showed that its presence allowed cells to restore the capability of wound closure and ameliorate redox status evaluated through the GSH/GSSG ratio and the total antioxidant capacity, hence partially restoring the resting conditions that were negatively affected by the presence of the oxidative imbalance that is H_2_O_2_-induced. It has been reported that in cells and in pathological animal models pretreated with AG, it is able to act as an antioxidant and a free radical scavenger [64]. 

In our experimental model, we did not observe any significant effect on redox status when cells were treated with AG in combined treatment with H_2_O_2_ (data not shown). Probably in the case of co-incubation, AG is not able to exert its antioxidant properties. It instead remains efficient in amine oxidase inhibition so that spermidine can exert its biological action through a mechanism that still needs to be investigated in detail.

The analysis of polyamine levels showed that H_2_O_2_ treatment caused a decrease in total PA content, making us speculate on the possibility that the negative effect of H_2_O_2_ on proliferation could also be explained by its influence on polyamine homeostasis. As confirmed by data from the literature, the PAs’ requests are higher during times of proliferative activity and the induction of polyamine biosynthesis precedes both nucleic acid and protein synthesis [65]. Moreover, various growth factors and hormones require the activation of the polyamine biosynthesis pathway to be effective in muscle cell proliferation and differentiation [65].

Interestingly, the PA reduction induced by H_2_O_2_ treatment was counteracted by the combined treatment with Spd-AG that also restored the capacity of wound closure.

These observations on PA content led us to investigate the influence of the treatments on polyamine gene expression. The analysis at the transcriptional level showed that H_2_O_2_ treatment affects the expression profile of polyamine metabolism genes. Interestingly, H_2_O_2_ leads to an increase in the transcription of ODC, SAT1 and SMOX. Those findings are in agreement with previous in vitro studies describing that H_2_O_2_ was able to induce polyamin catabolic gene expression [66,67] through the activation of the transcription factor Nrf2, and in the case of SAT1, also by NF-kB. Both transcription factors exert a vital function in cell and tissue protection versus oxidative stress damage [68,69,70,71].

Moreover, it was demonstrated that H_2_O_2_ produced by SMOX was able to induce an Nrf-2 response [37]. In this condition, H_2_O_2_ treatment induces SMOX expression, exacerbating H_2_O_2_ intracellular content and possibly leading to Nrf-2 increase as an antioxidant response. The addition of Spd-AG ameliorates the redox status and the total antioxidant capacity is able to revert the detrimental effects of H_2_O_2_, eluding the induction of SAT1 and SMOX.

We can speculate that, possibly due to a compensatory mechanism, H_2_O_2_ in an attempt to restore the polyamine level-increased gene expression and possibly their activities, causing a further increase in H_2_O_2_ production as a result of the enzyme activities, leading to a more pronounced redox imbalance.

However, despite the increase in gene expression of polyamine metabolic enzymes, the PA level did not increase after H_2_O_2_ treatment but instead decreased. This result could be due to an increase in SMOX and SAT1 enzymatic activity that occurs in polyamine oxidation. It can also be speculated that the increase in SAT1 expression could lead to an increased export of the PAs in the acetylated form so depriving cells of these important molecules essential for their survival. In fact, it has been reported that the conditional induction of SAT1 activity is sufficient to both lower PA pools, especially Spd, and inhibit cell growth by the increase of polyamine acetylated forms [72].

Interestingly, the addition of Spd-AG together with H_2_O_2_ treatment limited the increase of both SMOX and SAT1 expression, therefore maintaining the physiological condition.

As a limitation of the study, we did not perform enzymatic activity assays or spermidine transport across cell membranes. Further study will clarify these topics.

Altogether, the data of this study provides important new results regarding the complex process of regeneration of skeletal muscle, mediated by satellite cells. A detailed knowledge of this mechanism is essential for the understanding of the pathological conditions that lead to the degeneration of skeletal muscle and for the identification of new targeted therapeutic strategies. In this respect, Spd is revealed to be a protective molecule against oxidative stress insults and is capable of aiding the muscle regeneration processes.

## 4. Materials and Methods

All chemical reagents, unless otherwise specified, were purchased from Sigma-Aldrich Chemical (St. Louis, MO, USA).

### 4.1. Cell Cultures

C2C12 myoblasts (2 × 10^3^/cm^2^; ATCC, Manassas, VA, USA) were cultured in 25 cm^2^ culture flasks with Dulbecco’s-modified Eagle’s medium (DMEM; HyClone, Oud-Beijerland, The Netherlands) supplemented with Glutamax-I (4 mM l-alanyl-l-glutamine), 4.5 g/L glucose (Invitrogen, Carlsbad, CA, USA) and 10% heat-inactivated fetal bovine serum (Hy-Clone, Oud-Beijerland, The Netherlands). No antibiotics were used. The cells were incubated at 37 °C with 5% CO_2_ in a humidified atmosphere. Cells were split 1:6 twice weekly and fed 24 h before each experiment [73,74]. 

Cell viability was assessed by the trypan blue exclusion assay [75]. Briefly, C2C12 was seeded at a low density in 25 cm^2^ culture flasks and, after 24 h (≈20% confluence), was treated with different H_2_O_2_ (0.1–1 mM) and spermidine concentrations (1–100 μM) for 24 h (dose-dependence) and different H_2_O_2_ times (6–48 h, time-dependance). The maximum confluence reached by the cells was 75%.

For combined treatments, H_2_O_2_ 0.3 mM and spermidine 10 μM concentrations were chosen according to the above-mentioned assays. Aminoguanidine (AG, 1 mM) was used in combination with Spd according to Holbert and collaborators’ instructions in order to avoid Spd toxicity [45].

At the end of the treatments, cells were either harvested or collected by centrifugation (1200× *g* for 10 minutes at RT), and cell viability was assessed in each by trypan blue (0.05% *v*/*v* solution in PBS) exclusion mixed in a ratio of 1:1. Cell number counting was assayed by a hemocytometer, and cells excluding trypan blue dye (alive) or colored (dead) were counted. Each experimental point was repeated in triplicate. Results are expressed as the number of cells. Experiments were performed in triplicate with different cell preparations.

### 4.2. Trolox^®^ Equivalents Antioxidant Capacity

C2C12 myoblasts Trolox^®^ equivalents antioxidant capacity was evaluated spectrophotometrically, as previously described [76,77]. This assay evaluates the ability of cell lysates in preventing ABTS+ radical formation, compared to Trolox^®^ (vitamin E analogue) standards. Briefly, 10 μL of cell lysates or Trolox^®^ standards (0.125–2.0 mM) were incubated in ABTS-met-Myo-PBS buffer and the absorbance at 734 nm was monitored for 2 min.

The reaction was started by the addition of H_2_O_2_ (0.45 mM), followed for 10 min, and the variation of absorbance was then recorded. The variation of absorbance detected was compared to those obtained using Trolox^®^ standards. Cell lysate TACs were expressed as micromole Trolox equivalents/mg of protein tested. 

### 4.3. Polyamines Content 

Polyamine levels [putrescine (Put), spermidine (Spd), spermine (Spm)] were determined by HPLC with fluorimetric analysis [70,78]. After each treatment, cells were washed twice with PBS and then collected and centrifuged (1300× *g* for 10 min). Perchloric acid suspension (5%), supplemented with 1.7-diaminoeptane 100 µM as an internal standard, was added to the cell pellets. Samples were then sonicated in ice with Sonic Vibra-Cells to disintegrate the tissue and centrifuged at 16,100× *g* for 10 min. The supernatant was mixed with saturated Na_2_CO_3_ and then with acetone Dansyl chloride solution (7.5 mg/mL). The mixture was incubated overnight and protected from light at room temperature. The next day, the samples were centrifuged at 16,100× *g* for 15 min at 4 °C and proline solution (5%) was added to the supernatant to remove the unreacted Dansyl chloride. After 30 min, PAs were extracted with toluene (100%) with vigorous vortexing and then rested for 5 min at room temperature in the dark. The organic phase was dried in a 3 Speedvac Concentrator (Savant Instrument, Inc., New York, NY, USA). The dried Dansyl derivatives were stored at −20 °C or dissolved in methanol and immediately assayed. A high-performance liquid chromatography technique using the Agilent 1050 system (Agilent Technologies, Santa Clara, CA, USA) was used to detect the PA content with an Agilent 1050 photodiode type detector. Continuous on-line quantification of chromatographic peaks was carried out by the fluorimeter Agilent 1200 Spectra-Physics Model SP 4290 and the computing program software “Agilent Chem Station”. The separation of Dansyl derivatives was performed on C18 Hypersil BDS 250 mm × 4.6 mm at a constant room temperature of 22 °C ± 1. Two mobile phases were used: (A) water/acetonitrile/methanol (50%:30%:20%) and (B) acetonitrile/methanol (60%:40%), with the following elution program: 0–5 min: 72% A–28% B; 5–47 min: 72% A–28% B; 47–50 min: 36% A–64% B; 50–55 min: 20% A–80% B; 55–56 min: 15% A–85% B; 56–75 min: 72% A–28% B at a flow rate of 1 mL/min. PA content was normalized using protein concentration measured with the Bradford method; the amount of endogenous PA content was expressed as nmol/mg protein.

### 4.4. Wound Closure Assay

The ability of myoblasts to replicate and migrate in the culture plate under the different experimental conditions was evaluated by the “Wound Closure Assay”, as previously described [46] with some modifications. Briefly, C2C12 myoblasts (2 × 10^3^/cm^2^) were cultured in 6-well plates until approximately 50% confluence was reached.

Then, in a sterile environment, using a 1000 μL pipette tip, a vertical wound (1 mm) down through the cell monolayer was made by pressing firmly against the tissue culture plate.

Culture media and cell debris were carefully aspirated, and then fresh media was added. An initial picture was taken (time = 0). After two hours of recovery time, cells were treated with AG (1 mm), Spd (10 μM), H_2_O_2_ (0.3 mM) and combined treatments for a successive 24 h period. Cells were then placed under an inverted microscope and the wound closure was checked and snapshot pictures were taken.

In order to evaluate the wound closure from the picture acquired, the distance between the sides of the wound was measured (in triplicate) using ImageJ software (Rasband, W.S., ImageJ, U.S. National Institutes of Health, Bethesda, MD, USA).

### 4.5. Glutathione Homeostasis 

Intracellular reduced (GSH) and oxidized (GSSG) glutathione contents were quantified by a DTNB–glutathione reductase recycling assay, as previously described [79]. 

Briefly, 10^7^ cells were collected and suspended in (1:1) (*v*/*v*) μL 5% sulfosalicylic acid (SSA). Cells were lysed by freezing and thawing three times and then were centrifuged at 10,000× *g* for 5 min at +4 °C. The deproteinized supernatant was then analyzed for total glutathione content. Oxidized glutathione (GSSG) was selectively measured in samples where reduced GSH was masked by pretreatment with 2-vinylpyridine (2%) [80]. A total of 10 μL of the sample was added to the reaction buffer [700 μL NADPH (0.3 mM), 100 μL DTNB (6 mM), 190 μL H_2_O]. The reaction was started by adding 2.66 U/mL glutathione reductase and followed at 412 nm by the TNB stoichiometric formation. The variation of absorbance detected was compared to those obtained by using glutathione standards, and the results were normalized for protein content.

### 4.6. RNA Isolation, Reverse Transcription and qReal-Time PCR

Total RNA from C2C12 cells was extracted using TRIzol Reagent (Invitrogen, Carlsbad, CA, USA) and retrotranscribed into cDNA in two steps by the SuperScript III First-Strand Synthesis System (Invitrogen, Carlsbad, CA, USA) according to the manufacturer’s instructions [81]. Cell fractionation was carried out using the Ambion PARIS kit according to the manufacturer’s protocol. The RNase R treatment was performed as follows: 2 µg of total RNA was treated with 2u RNase R/µg (MRNA092, Epicentre Biotechnologies–Madison, WI, USA) for 15 min at 37 °C and purified by phenol chloroform extraction. Primers used for the PCR amplification of specific genes are shown in Table 1. For RT-PCR amplification, specific primers used for ornithine decarboxylase (ODC), spermine-/spermidine-N1-acetyltransferase 1 (SSAT1), polyamine oxidase (PAOX), spermidine synthase (SDS), spermine synthase (SMS) and spermine oxidase (SMOX) genes are listed in Table 1. PCR reactions were performed in a 50 μL reaction volume using the DreamTaq DNA Polymerase (Thermo Fisher Scientific, Waltham, MA, USA) Reaction Kit in accordance with the manufacturer’s protocol. Amplification and digested products were analyzed by 1 and 2.5% agarose gel electrophoresis according to the expected fragment molecular weight. qRT-PCR analyses were carried out by the SYBR-Green method, and the corresponding specific primers are listed in Table 1. Reactions were performed in the AriaMx Real-Time PCR System (Agilent Technologies) using the following program: 40 cycles of 95 °C for 2 min, 95 °C for 5 s and 60 °C for 30 s. The mRNA for the constitutive glyceraldehyde-3-phosphate dehydrogenase (GAPDH) was examined as the reference transcript. GAPDH was chosen as the reference gene because its expression did not change at the different experimental points. The data are calculated relative to the internal housekeeping gene according to the second derivative test [delta–delta Ct (2−ΔΔCT)] method [6].

### 4.7. Statistical Analysis

We used Prism software (GraphPad Software, San Diego, CA, USA) to check for statistical significance. The results are presented as means ± SEM of three independent experiments, each performed in triplicate. Statistical analysis was performed by the one- or two-way analysis of variance (ANOVA), followed by the Bonferroni’s multiple comparison test according to the evaluation of the statistical differences from samples to the control or between all the samples with each other, respectively. *p* < 0.05 was accepted as significant. 

## 5. Conclusions

The present study demonstrated that the treatment of C2C12 myoblasts with spermidine in combination with AG increased cell number and accelerated scratch wound closure, while treatment with Spd alone caused dose-dependent cytotoxicity. The oxidative environment induced by H_2_O_2_ exposure caused redox status imbalance and cell death. The combined treatment with Spd-AG showed antioxidant effects on C2C12 myoblasts, partially restoring cellular total antioxidant capacity, GSH/GSSG ratio and an increase in cell viability through a reduction in cell death. Moreover, Spd-AG counteracted the polyamine content decrease observed by H_2_O_2_ treatment alone, most probably by the impairment of the catabolic PA transcripts SMOX and SAT1 induction resulted from H_2_O_2_ administration.

## Figures and Tables

**Table 1:** Primers used for the study.

Gene	Forward ^1^	Reverse ^1^
ODC	5′-GAAGAGATCACCAGTGTAATC-3′	5′-CTCATCTTCATCGTCAGAGC-3′
PAOX	5′-GGGAAGATACATCGCCCTTA-3′	5′-GGACCAAAAATCCAATGAGC-3′
SMOX	5′-ACTCCAAGAATGGCGTGGC-3′	5′-CGACGCTGTTCTGACTCTC-3′
SMS	5′-ACAAGAATGGCAGCTTTGCC-3′	5′-GAACTATGGGTGGTAATCGC-3′
SDS	5′-CGGAAGGTGCTGATCATCG-3′	5′-TCGCCCACGTGGAGAGT-3′
SAT1	5′-CACTGGACCCCTGAAGGTTA-3′	5′-CAGCAACTTGCCAATCCATG-3′
GAPDH	5′-GGTTGTCTCCTGCGACTTC-3′	5′-GGTGGTCCAGGGTTTCTTAC-3′

^1^ qRT-PCR.

## Data Availability

The data presented in this study are available on request from the corresponding author.
